# Soluble CD83 improves and accelerates wound healing by the induction of pro-resolving macrophages

**DOI:** 10.3389/fimmu.2022.1012647

**Published:** 2022-09-30

**Authors:** Dmytro Royzman, Katrin Peckert-Maier, Lena Stich, Christina König, Andreas B. Wild, Miyuki Tauchi, Christian Ostalecki, Franklin Kiesewetter, Stefan Seyferth, Geoffrey Lee, Sabine A. Eming, Maximilian Fuchs, Meik Kunz, Ewa K. Stürmer, Eva M. J. Peters, Carola Berking, Elisabeth Zinser, Alexander Steinkasserer

**Affiliations:** ^1^ Department of Immune Modulation, University Hospital Erlangen, Friedrich-Alexander-Universität Erlangen-Nürnberg (FAU), Erlangen, Germany; ^2^ Department of Internal Medicine 2, University Hospital Erlangen, FAU, Erlangen, Germany; ^3^ Department of Dermatology, University Hospital Erlangen, FAU, Erlangen, Germany; ^4^ Division of Pharmaceutics, Department of Chemistry and Pharmacy, Friedrich-Alexander University Erlangen-Nürnberg (FAU), Erlangen, Germany; ^5^ Department of Dermatology, University Hospital Cologne, Center for Molecular Medicine Cologne (CMMC), Cologne Excellence Cluster Cluster of Excellence for Aging Research (CECAD), University of Cologne, Cologne, Germany; ^6^ Fraunhofer Institute for Toxicology and Experimental Medicine (ITEM), Hannover, Germany; ^7^ Department of Medical Informatics, Friedrich-Alexander University Erlangen-Nürnberg (FAU) and University Hospital Erlangen, Erlangen, Germany; ^8^ Department for Vascular Medicine, University Medical Center Hamburg-Eppendorf, Hamburg, Germany; ^9^ Psychoneuroimmunology Laboratory, Klinik für Psychosomatik und Psychotherapie, Justus-Liebig Universität Gießen, Gießen, Germany

**Keywords:** CD83, wound healing, macrophages, therapy, tissue regeneration, stem cells

## Abstract

To facilitate the recovery process of chronic and hard-to-heal wounds novel pro-resolving treatment options are urgently needed. We investigated the pro-regenerative properties of soluble CD83 (sCD83) on cutaneous wound healing, where sCD83 accelerated wound healing not only after systemic but also after topical application, which is of high therapeutic interest. Cytokine profile analyses revealed an initial upregulation of inflammatory mediators such as TNFα and IL-1β, followed by a switch towards pro-resolving factors, including YM-1 and IL-10, both expressed by tissue repair macrophages. These cells are known to mediate resolution of inflammation and stimulate wound healing processes by secretion of growth factors such as epidermal growth factor (EGF) and vascular endothelial growth factor (VEGF), which promote vascularization as well as fibroblast and keratinocyte differentiation. In conclusion, we have found strong wound healing capacities of sCD83 beyond the previously described role in transplantation and autoimmunity. This makes sCD83 a promising candidate for the treatment of chronic- and hard-to-heal wounds.

## Introduction

Injuries disrupt the protective function of the skin barrier and enhance the susceptibility to infections. Therefore, a fast wound healing process is crucial for maintaining the physical integrity of the skin.

Mechanistically, wound healing is a complex dynamic process, which consists of four highly integrated phases: (I) homeostasis, (II) inflammation, (III) reepithelization, and (IV) tissue remodeling. These phases are controlled by multiple cell types that secrete specific growth factors, cytokines, and chemokines (1). Upon injury, platelets attenuate blood flow by coagulation and induce the inflammatory phase by the release of α-granules (2). In this phase, neutrophils, macrophages and lymphocytes prevail the wound area and establish a pro-inflammatory cytokine milieu. Subsequently, macrophages (MΦ) undergo a transition from a pro-inflammatory to a pro-reparative phenotype, thereby facilitating resolution of inflammation and the progress towards the third phase, i.e. the proliferation phase by the secretion of anti-inflammatory mediators such as IL-10 and growth factors such as TGF-β and VEGF (3, 4). In this phase, the secretion of VEGF induces and stabilizes both angiogenesis and lymphangiogenesis, by an enhanced proliferation and migration of pluripotent mesenchymal cells which ensures sufficient supply with nutrients to the healing tissue. In the final stage, tissue homeostasis is restored and the inflammatory cells either exit the site of injury or undergo apoptosis.

Regulation of inflammation is crucial to the healing process and molecules that can promote the reestablishment of homeostasis are of high medical interest. CD83 is a member of the Ig superfamily and expressed primarily on a wide variety of activated leukocytes, including dendritic cells (DCs), macrophages MΦ, T- cells, B cells and regulatory T cells (Tregs), as well as on thymic epithelial cells (TEC) (5, 6). So far, two CD83 isoforms have been described. The membrane-bound form of CD83 (mCD83) expressed by TECs is absolutely essential for the positive selection and development of CD4^+^ T cells in the thymus (7). In addition, our group recently reported that mCD83 expression by Tregs is crucial for their development and that the specific deletion of CD83 on Tregs leads to exacerbated autoimmune pathologies and impaired resolution of inflammation (8, 9). Furthermore, specific conditional KO CD83 in DC leads to excessive inflammatory immune responses and drastically increased autoimmunity (10). The soluble isoform of CD83 (sCD83) consists of the extracellular domain (6), and using recombinant sCD83, we and others have shown that sCD83 profoundly modulates inflammatory immune responses by blocking pro-inflammatory cytokine expression and strongly inducing anti-inflammatory mediators (11–14). In addition, sCD83 leads to the activation and expansion of Tregs in an indoleamine-2, 3-dioxygenase (IDO) dependent manner (12, 15). Mechanistically, sCD83 has been reported to bind to the Toll-like receptor (TLR) 4/MD2-complex, subsequently altering the pro-inflammatory signaling cascade towards an anti-inflammatory and pro-resolving phenotype via the induction of potent immune modulators, such as IDO1, TGF-β and IL-10 (13). These features make CD83 a very interesting candidate for immune regulation in wound healing.

Under pathophysiological conditions, including diabetes, venous or arterial diseases, and metabolic deficiencies of old age skin, repair processes are disrupted (1, 16, 17). In addition, smoking and malnutrition contribute to wound healing complications (18). Furthermore, wound healing is impaired by immunosuppressive drugs and glucocorticoids, commonly used upon transplantations or the treatment of autoimmune disorders (19). Encouraged by our previous observation that the CD83 molecule is an important checkpoint for resolution of inflammation and induction of regenerative processes (6), we hypothesized that CD83 is an interesting candidate for the modulation and acceleration of wound healing processes. Since specific mediators, such as cytokines, growth factors and TLR-mediated signaling events play a crucial role during wound healing, we analysed if sCD83 could modulate these regenerative processes *in vivo*. This approach confirmed that sCD83 induced and accelerated high quality wound healing in skin, both when applied systemically and topically. Thus, the present work demonstrates for the first time that sCD83 has proregenerative potential for the treatment of wounds.

## Methods

### Mice

Female C57BL/6 mice were purchased from Charles River Laboratories (Sulzfeld) and maintained under pathogen-free conditions according to the institutional and national guidelines for the care and use of laboratory animals.

### Experimental design of the full-thickness excisional wound model and analyses of wound closure

For surgical procedures, mice were anesthetized using a mixture of ketamine and xylazine (120 mg/kg and 20 mg/kg body weight, respectively). Hair at the dorsum back were scrubbed when required for experimental setup before making the incisions. To investigate the role of sCD83 in physiological tissue responses, 6-mm circular full skin thickness excisional wounds were generated by punch biopsy (pfm medicals) on the dorsum of C57BL/6 mice (7-8 weeks of age). In order to facilitate topical administration and prevent wound healing by contraction, 8mm silicone rings (Thermo scientific) were mounted around the wound area, using vetbond (3M), when required. Wounds were regularly photographed and wound size assessed by caliper (length (L) and width (W)) and calculated based on wound size relative to the initial wound dimension. Wound area (WA) on day X (dX) and wound closure (% of baseline) was calculated by the following equations:


Wound area(WA)=(L/2)∗(W/2)∗π

Wound closure (%)=(WA d0–WA dX)/WA d0∗100

A daily dose of 100 µg sCD83 or the corresponding volume of PBS (mock control) was administered i.p. from day 0 until day 7. sCD83 was produced as already described (15). Biopsies were obtained as 8 mm punches (pfm medicals) around the former wound area on day 3, 6, 7, and 12 and samples were either fixed in 4% paraformaldehyde (PFA) for histological analyses, snap frozen in liquid nitrogen for protein extraction, or stored in RNAlater® (Thermo Fisher Scientific) at -80°C for subsequent RNA-analyses. For topical sCD83 application, dorsal hair were depilated to ensure a uniform deposition of the hydrogel-stabilized sCD83 protein. For this purpose, mice were anesthetized, and 25 µg sCD83-hydrogel were topically applied on day 1, 3, and 5.

### Isolation of bone marrow-derived precursor cells and generation of MΦ

Hind limbs were removed from 8 weeks old female C57BL/6 mice. Femur and tibia were flushed with 10 ml PBS and BMC were collected in sterile 6 cm petri dishes. Cells were transferred into 15 ml falcon tubes and centrifuged at 1,200 rpm for 5 min (4°C). 2 x 10^6 BMCs were seeded in a 6 cm petri dish containing 3 ml D10 medium (composition: DMEM (Lonza), 10% FCS (Merck, Germany), penicillin-streptomycin-glutamine solution (Sigma Aldrich), and 50 µM β-mercaptoethanol). After adherence, bone marrow precursor cells (3x10^6^) were seeded in D10 medium supplemented with 10% of M-CSF supernatant generated from L929 cell line, kindly provided by Prof. Dr. Jochen Mattner at the University Hospital Erlangen; Department of Microbiology [~500 pg/ml; as determined by M-CSF-specific ELISA (R&D Systems, DY416)]. The sCD83 protein (25 µg/ml) was administered during the MΦ differentiation process on day 1 and day 3. A corresponding volume of PBS was added to mock controls. At day 6 cells were harvested, washed in fresh D10 medium and subsequently seeded in 24 well plates at a density of 2*10^6 per ml. MΦ were either stimulated with IL-4 (40 ng/ml) or left untreated for 16 hours. mRNA expression analyses of MΦ were performed by qPCR.

### Administration of Mϕ to wounds

Full-thickness excisional wounds through the dorsal skin were induced as described above. MΦ, which were generated in the presence of sCD83 or PBS, were applied directly onto the wound beds in a concentration of 1,5×10^6^ MΦ in 25 µl PBS per wound. PBS only wounds served as control. In order to prevent rubbing or leaking a 8 mm silicone ring was glued around every single wound using Vetbond (M3) and further covered with Tegaderm™ transparent dressing (3M). Pictures were taken on day 3 and wound size was determined by ImageJ software relatively to the size of the 8 mm silicone rings.

### Preparation of hydrogel

Hydrogels containing sCD83 were prepared by the addition of 4% (w/v) hydroxyethylcellulose (Natrosol® 250 HX Pharm; Caelo) to sCD83 solutions. The hydroxyethylcellulose was dispersed on the surface of the aqueous sCD83 solutions and allowed to gel overnight. The PBS mock samples were prepared identically.

### Cell extraction from skin biopsies

Mice were euthanized by CO_2_ inhalation or cervical dislocation at day 3 or 6. Skin biopsies of 8 mm diameter were isolated and cut into small pieces using a surgical scalpel, and transferred into a 1.5 ml tube containing 1 ml DMEM/F12 (Thermo Fisher Scientific), mixed with Liberase TL (Sigma Aldrich), in a final concentration of 0.26 WU/ml. Samples were then incubated for 30 min at 37°C and 700 rpm on a shaking incubator. Afterwards, the suspension was filtered through a 70 µm cell strainer using the plunger top of a 5 ml syringe. The strainer was flushed with 5 ml DMEM/F12 and the flow through was centrifuged for 5 min (300 g at 4°C). After an additional washing step with 5 ml PBS (5 min, 300 g at 4°C), the pellet was resuspended in 1 ml PBS, filtered through a 35 µm cell strainer, and used for subsequent surface staining and flow cytometry.

### Flow cytometry

Cell samples, isolated from skin biopsies, were centrifuged for 3 min (500 g at 4°C), and the cell pellet was resuspended in 50 µl PBS (supplemented with 1 mM EDTA), containing fluorescence-coupled antibodies purchased from Biolegend, unless otherwise stated, directed against CD45 (PerCP; Biolegend; # 103129/clone 30-F11), CD3 (BV421; # 100228/clone 17A2), CD11b (PE-Cy7; # 101215; clone M1/70), B220 (PE; BD; # 553080; clone RA3-6B2), F4/80 (FITC; #123108/clone BM8), Ly6C (APC-Cy7; # 128026/clone HK1.4)), Ly6G (APC; # 127614/clone 1A8)), and LIVE/DEAD Fixable Aqua DEAD Cell stain (Thermo Fisher Scientific). All antibodies were purchased from BioLegend, except B220 (BD). After incubation for 30 min at 4°C, cells were washed with 500 µl PBS (500 g, 3 min at 4°C), resuspended in 100 µl PBS, and assessed using the FACS Canto II flow cytometer (BD).

### RNA extraction, cDNA synthesis and qPCR analyses

Biopsies were collected and stored in RNAlater® at -80°C. For RNA isolation, samples were thawed gently on ice and homogenization was performed in 400 µl RLT buffer (supplemented with 1% β-mercaptoethanol), using innuSPEED Lysis Tube I and SpeedMill PLUS (both from AnalytikJena). After three homogenization steps, each 20 sec with 5 min cooldown at -20°C, the suspension was centrifuged for 3 min at 16,000 g and the supernatant was collected for RNA extraction, using the RNeasy Plus Mini Kit (Qiagen), according to manufacturer’s protocol. cDNA synthesis was performed according to the First Strand cDNA Synthesis Kit manual (Thermo Fisher). Ribosomal protein L4 (*Rpl4*) was used as housekeeping gene. The polymerase chain reaction and quantifications were performed using the CFX96 Touch (Bio-Rad). Nucleotide sequences of the primer pairs are provided in the **Table 1**.

**Table 1:** Primer used for RT-PCR.

Gene	Sequence forward	Sequence reverse
*Dkk-1*	AAACCTTGGTAATGACCACAACG	AGAAGTGTCTTGCACAACACA
*Egf*	GGAAGCCACGCTTACATTCAT	ACTGAGTAGAAGATCCGATCACC
*Il-10*	CCAAGCCTTATCGGAAATGA	TTTTCACAGGGGAGAAATCG
*Il-1β*	TGCCACCTTTTGACAGTGATG	ATGTGCTGCTGCGAGATTTG
*Lef-1*	GCCACCGATGAGATGATCCC	TTGATGTCGGCTAAGTCGCC
*Mmp13*	TCCCTGGAATTGGCAACAAAG	GGAATTTGTTGGCATGACTCTCA
*Rpl4*	GCTGAACCCTTACGCCAAGA	TCTCGGATTTGGTTGCCAGT
*Slpi*	GGCCTTTTACCTTTCACGGTG	TACGGCATTGTGGCTTCTCAA
*Tnf-α*	GTGATCGGTCCCCAAAGGG	CCAGCTGCTCCTCCACTTG

### Bioinformatics

RNA was isolated from day 3 wound biopsies of sCD83- or mock-treated mice as already described. An additional DNase digestion step was performed in order to remove residual DNA. RNA sequencing was performed by Novogene and analyzed as described:

Raw paired-end reads were aligned to the reference genome (GRCh38) using Rsubread (v 2.6.4) (20) within R programming language (v 4.1.1). Quantification was performed using featureCounts function from RSubread. After exploration of data, two samples were identified as definite outliers and excluded from the analysis. Differential expression analysis was performed following the previously published pipeline (21) using DESeq2 (v 1.32.0) (22). g:Profiler web-tool (23) was used for functional analysis of differentially expressed genes.

### Histology

Skin biopsies were obtained as 8 mm punches around the former wound area on day 3, 6, and 7, respectively. Excised tissues were fixed in 4% formalin solution for five days. All tissues were processed using conventional histochemical techniques, embedded in paraffin wax and then sectioned at a 5 μm thicknesses, mounted onto glass slides, de-paraffinized and stained with hematoxylin and eosin (HE). Alternatively, cutaneous wound slides were stained to detect CD31 expression, using a rabbit monoclonal anti-CD31 antibody (dilution 1:3,000; abcam; # ab182981; clone: EPR17259).

### Multi-epitope ligand cartography technique

Skin biopsies (8 mm) were isolated on day 6 and mounted in Tissue-Tek® O.C.T™ (Sakura) in Tissue-Tek® Cryomold® and stored at -80°C. Five µm slices were used for immunofluorescence imaging using the MELC-technique with the following antibodies: Ly6C (FITC; BD, # 561085; clone: AL-21), Ly6G (PE; # 551461; clone: 1A8), CD117 (PE; eBioscience; # 12-1171-82; clone: 2B8), Ki67 (FITC; Miltenyi Biotec; # 130-117-803; clone: REA183), CD31 (FITC; Biolegend; # 102405; clone: 390), cytokeratin-14 (CK, FITC; abcam; # ab77684; clone: LL002) and propidium iodide (PI; Genaxxon bioscience; M3181.0010). Sample preparations from tissue, data generation, and analysis were performed as described previously (24). ImageJ software was used for evaluation.

### Statistical analysis

All data are expressed as mean ± SEM. Normal distribution was verified by Shapiro-Wilk test and statistical significance was calculated using unpaired multiple t-test for n=3 or the Mann-Whitney U test (n>3) for nonparametric distribution. Grouped data were analyzed using One-way or Two-way ANOVA by multiple comparison and a post-hoc correction according to Sidak. All calculations were performed using GraphPad Prism 8 (GraphPad). P-values< 0.05 were considered significant.

## Results

### Systemic administration of sCD83 accelerates cutaneous wound healing

**Figure 1:**
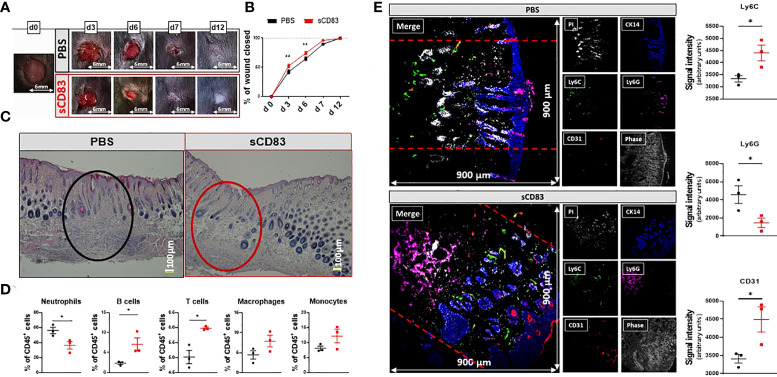
sCD83-treatment accelerates wound healing and modulates cellular composition within the wound beds. Wounds were inflicted in anesthetized 7-weeks-old female C57BL/6 mice using a 6 mm biopsy punch. Administration of 100 µg sCD83 was performed systemically until day 7 on a daily basis. **(A)** Representative images of wound areas on day 0, 3, 6, 7, and 12. **(B)** Wound size was determined using caliper and expressed as percentage of wound closure relatively to the day 0 value (each group and time point n≥12). **(C)** Representative H&E slides of day 7 wound biopsies. **(D)** Cellular composition within the wound biopsies of sCD83- and mock-treated mice, as assessed by flow cytometric analyses of day 6 samples (n=3). **(E)** Representative MELC-images (900µm x 900µm) of day 6 biopsy samples from PBS- and sCD83-treated mice [**(E)**; left-hand side] along with the corresponding quantification of signal intensity as determined using ImageJ with n=3 [**(E)**; right-hand side]. Dashed lines mark the wound site. Data are illustrated as mean ± SEM. Statistical analyses: **(B)** Two way ANOVA. Asterisks mark statistically significant difference (*p < 0.05 and **p < 0.01). The absence of asterisks indicates that there is no statistical significance.

To assess the impact of sCD83 treatment on wound healing, 6 mm punch and biopsy wounds were inflicted to mice and monitored every other day for 12 days. Daily systemic administration of 100 µg sCD83, as titrated in prior studies, was applied from day 0 to day 7 and resulted in an accelerated wound closure when compared with PBS-treated control mice, as shown in **Figures 1A**,**B** (25). While in PBS-treated control mice complete wound closure was only reached on day 12, this stage was already reached in sCD83-treated mice on day 7, in evidence of a dramatically improved wound healing process compared to normal wound healing. Histopathologically, an almost completely restored skin architecture was observed in hematoxylin- & eosin-stained skin sections obtained on day 7 of sCD83 treatment, while the wound area of PBS-treated controls showed diffuse cell clusters, i.e., granulation tissue (**Figure 1C**). Preceding this, a significant depletion of neutrophils was detected within the wound beds on day 6 in sCD83-treated mice by flow cytometry, shortly before wound healing was complete, while T cell and B cell numbers increased significantly and a prominent accumulation of MΦ and monocytes was detected (**Figure 1D**). This difference in the composition of immune cells surrounding the wound area was not yet present on day 3 post wounding (data not shown) although wound healing was significantly accelerated on both days, 3 and 6. A representative gating strategy is depicted in **Supplementary Data 1**.

To further characterize the cellular distribution within distinct cutaneous compartments on day 6, i.e., epidermis, dermis and hypodermis, a Multi-Epitope-Ligand-Cartography (MELC)-technique was applied to map distinct cell populations and vascular structures within wound sites on day 6. Representative images of sCD83-treated mice revealed an emigration of Ly6G^+^ neutrophils from the dermis into the subcutaneous tissue (**Figure 1E**, lower panel, magenta signal), while Ly6C^+^ monocytes were increased in the dermis (**Figure 1E** lower panel, green signal), when compared to control mice (**Figure 1E**, upper panel). Moreover, capillary formation in sCD83-treated mice was increased on day 6, as indicated by a prominent staining for CD31, a marker for endothelial cells (**Figure 1E**, lower panel, red signal). Notably, cytokeratin 14 (CK14), an intermediate filament protein within the neo-epidermis (26), was prominently increased in sCD83-treated mice, indicating an accelerated differentiation process upon sCD83 treatment (**Figure 1E**, lower panel, blue signal).

### sCD83 drastically changes the transcriptome during early wound healing and boosts the regeneration process

**Figure 2:**
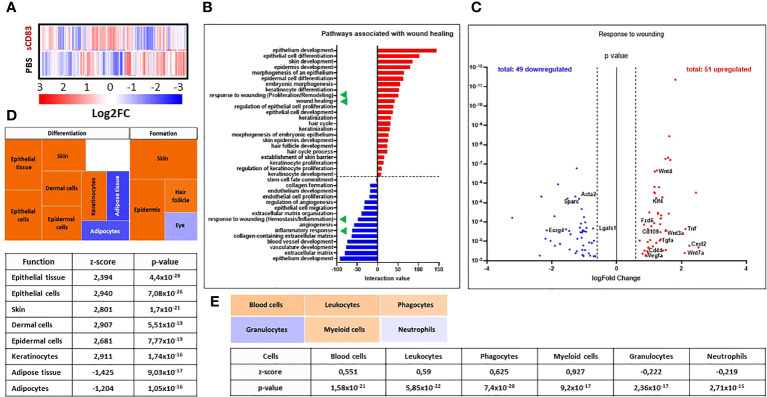
sCD83-treatment changes the transcriptome profile towards a wound healing phenotype. RNA was extracted from day 3 wound biopsies and used for RNA sequencing analyses. The transcriptome of sCD83-treated mice was compared to mock controls. **(A)** Representative heat map of differently expressed transcripts in wound biopsies of sCD83-treated and PBS-treated mice on day 3: induction = red and suppression = blue (each n=2). **(B)** Pathway analysis was performed based on the transcriptome analyses and its interaction value with the corresponding pathways (red = induced and blue = suppressed). Green triangles highlight two wound healing-associated pathways that were induced or suppressed by sCD83, respectively. **(C)** Volcano plot of RNA sequencing analyses of wound biopsies of sCD83- vs. PBS-treated mice on day 3 included in the response to wounding pathway. Dots depicted on the right-hand side of the logFold change 0 value represent significantly upregulated gene transcripts (red), while the dots on the left-hand side represent significantly downregulated transcripts (blue) with logFC ≥ 0.585, respectively. The Y-axis depicts the p value. **(D)** Transcriptome data were further analysed using the Ingenuity Pathway Analysis (IPA) software from QIAGEN’s revealing enhanced wound healing pathways in sCD83-treated mice. **(E)** Cellular composition within day 3 wound sites of sCD83-treated mice as assessed from the RNA sequencing data by IPA.

In order to learn, if prior to histologically detectable achievements in sCD83 treatment molecular and cellular mechanisms responsible for the observed pro-regenerative effects of sCD83, can be detected, RNA sequencing analyses were performed using RNA isolated from day 3 wound biopsies of sCD83- or PBS-treated mice. Although we did not observe any effects on sCD83-treatment on the cellular composition (data not shown), we assumed day 3 to be an interesting time point for transcriptome analyses, since it was the first time point showing significantly accelerated wound healing by sCD83. Indeed, the heat map of RNA sequencing analyses shown in **Figure 2A** summarizes the striking differences within the transcriptome of both groups on day 3. In total, 1095 gene transcripts were upregulated, while 979 were downregulated in biopsies derived from sCD83-treated animals (**Supplementary Data 2**). Subsequent pathway analyses revealed that sCD83 treatment affected wound healing-associated pathways early as day 3, summarized in **Figure 2B**. Among the downregulated pathways (blue) a strong inhibition of the inflammatory immune response was detected, while wound healing pathways were generally upregulated by sCD83 (red).

Interestingly, responses associated with “wounding” were present in both the upregulated and downregulated pathways (**Figure 2B**, green triangles). A deeper analysis of the respective responses revealed a suppression of transcripts that are normally associated with the early phase of wound healing, such as *Ecrg4*, which is responsible for neutrophil recruitment during the inflammatory phase of wound healing (**Figure 2C**) (27). Moreover, *Lgals1* and *Acta2* are associated with fibrotic wound healing and hypertrophic scar formation, which were not observed in sCD83-treated mice (28, 29). In sharp contrast, the upregulated gene transcripts included *Klf4*, *Cd109* along with genes of the Wnt/Frizzled cascade, indicating an accelerated turnover of stem cells (**Figure 2C**).

These observations were further substantiated using the Ingenuity Pathway Analysis (IPA) software from QIAGEN, which identified the induction of pathways associated with the proliferation and remodeling phases of wound healing, resulting in accelerated formation and differentiation processes in skin and skin compartments of sCD83-treated samples (**Figure 2D**; orange rectangles). Notably, hair follicle formation was also increased in samples of CD83-treated mice as indicated by the upregulation of e.g. *Gsdma3* and *Lhx2*, genes with a pivotal role for hair follicle differentiation and formation (data not shown) (30, 31). Of note, hair follicle formation is a phenomenon normally absent in small sized healing wounds (32). Thus, data derived from the IPA analyses support our hypothesis, that the treatment with sCD83 enhances regenerative pathways which increase wound healing properties and promote tissue regeneration. Moreover, transcript-pathway analyses using the IPA software also indicated the “replacement” of neutrophils and granulocytes (**Figure 2E**, blue rectangles) by leukocytes and myeloid-derived phagocytes in wound biopsies of sCD83-treated mice on day 3 by calculating cell quantities according to the transcriptome profiles (**Figure 2E**, light orange rectangles). This agrees very well with our flow cytometric analyses showing an increase in leukocytic and myeloid-derived cells and a decrease of neutrophils in wound biopsies of sCD83-treated mice (**Figure 1D**).

**Figure 3:**
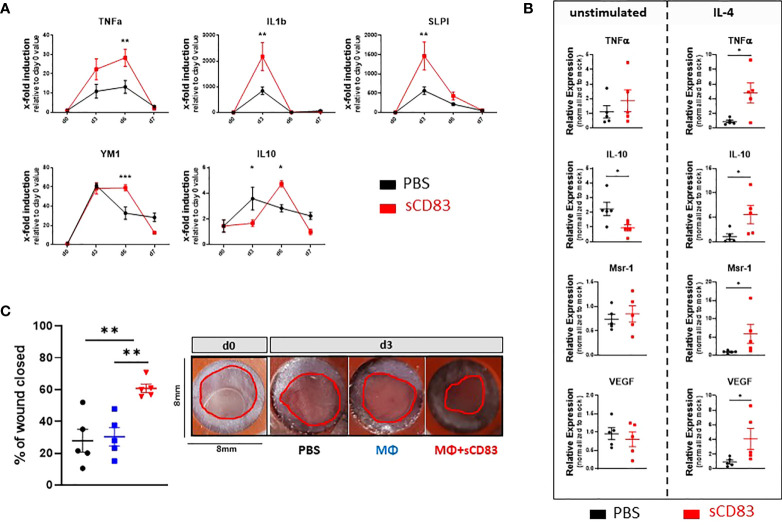
sCD83 promotes MΦ differentiation and activity in the course of wound healing. **(A)** Transcript analyses were performed with skin biopsies relatively to the day 0 value (each group and time point n≥4). **(B)** RNA induction levels of *Tnfα, Il-10, Msr-1* and *Vegf* were assessed in sCD83-treated MΦ under steady state conditions or in the presence of IL-4 (n=3) with the control group shown in black and sCD83-treated mice in red. **(C)** *Ex vivo* generated MΦ, cultured in the presence or absence of sCD83, were adoptively transferred into the wound beds and wound closure was assessed by caliper on day 3 relatively to the day 0 value (each n=5). Red circles highlight the wound area. **(A)** Two-way ANOVA. **(B)** Mann-Whitney test and **(C)** One-way ANOVA with F= 11,03. Asterisks mark statistically significant difference (* p< 0.05, **p < 0.01 and ***p < 0.001). The absence of asterisks indicates that there is no statistical significance.

Since the inflammatory phase of the wound healing process is initiated by cytokines, we investigated the expression patterns of TNFα and IL-1β as representative proinflammatory cytokines by RT-PCR analyses (33). An upregulation of IL-1β was observed on day 3 followed by a strong decrease on day 6 and 7 (**Figure 3A**). TNFα was found increased on day 3 and 6 but no longer on day 7 (**Figure 3A**). A very interesting molecule which plays a crucial role in the resolution of inflammation in wound healing is the secretory leukocyte protease inhibitor (SLPI) (34). As shown in **Figure 3A**, SLPI was significantly upregulated in samples of sCD83-treated mice on day 3 compared to PBS-treated controls. Noteworthy, SLPI is released by MΦ during the clearance of apoptotic debris, again supporting the role of regenerative MΦ for the sCD83-enhanced wound healing capacities (34). Indeed, YM-1, a marker for resolving MΦ, was stably induced throughout the wound healing process (day 3 until day 6) in sCD83-treated mice (**Figure 3A**). As representative molecules for the resolution of inflammation and thus pro-resolving MΦ, we have analyzed the expression pattern of IL-10 and TGFβ expression patterns. Upregulation of IL-10 expression was significantly delayed in sCD83-treated mice on day 3, but strongly upregulated on day 6 (**Figure 3A**). Interestingly, IL-10 levels declined on day 7, as did YM-1 levels (**Figure 3A**). No difference was observed in the expression of TGFβ between sCD83- and control-treated mice (data not shown).

### Macrophages play a crucial role for the sCD83 mediated accelerated wound healing

The above-described data suggested a possible role of alternatively activated pro-resolving MΦs in sCD83-treated mice at an early time point during the healing process. Thus, our next aim was to investigate whether sCD83 directly affects MΦ differentiation and activity. We generated MΦ from mononuclear cells isolated from the bone marrow of the hind limbs and incubated them with 25 µg/ml sCD83. However, we did not observe a significant impact of sCD83 on these MΦ (**Figure 3B**; left-hand site). In the following experiments we therefore added an important factor known to modulate the MΦ phenotype towards an alternative pro-resolving one, i.e. IL-4 (35). Strikingly, the stimulation of MΦ by IL-4 boosted sCD83-mediated effects regarding the expression of transcripts associated with resolving tissue damage and accelerating tissue repair MΦ, such as *Tnfα, Il-10, Msr-1* and *Vegf* (**Figure 3B**, right hand site) (36, 37).

Having shown the impact of sCD83 on MΦ activation *in vitro*, the next question was, whether sCD83 effects elicited in MΦ are responsible for the significant impact on cutaneous wound healing *in vivo*. To address this, we performed adoptive transfer experiments using either sCD83-preincubated MΦ, mock-treated MΦ, or PBS alone, into freshly punched 6 mm wound beds and tracked the wound closure until day 3. Of note, although the previous experiments showed that IL-4 increases sCD83 mediated effects on MΦ *in vitro*, we did not drive these cells into a pro-resolving phenotype before application *in vivo*. Moreover, no additional administration of IL-4 was required during wound treatment, since IL-4 accumulates shortly after wound infliction, within the natural wound healing environment (38). As shown in **Figure 3C**, the transfer of sCD83-treated MΦ had a strong and significant influence on wound healing already on day 3. In sharp contrast, untreated MΦ and PBS alone had no influence on wound closure (**Figure 3C**). These results clearly demonstrated that modulation of MΦ by sCD83 improves wound closure by boosting and accelerating the natural remodeling kinetics.

### sCD83 promotes epithelial stem cell activity during wound healing processes

**Figure 4:**
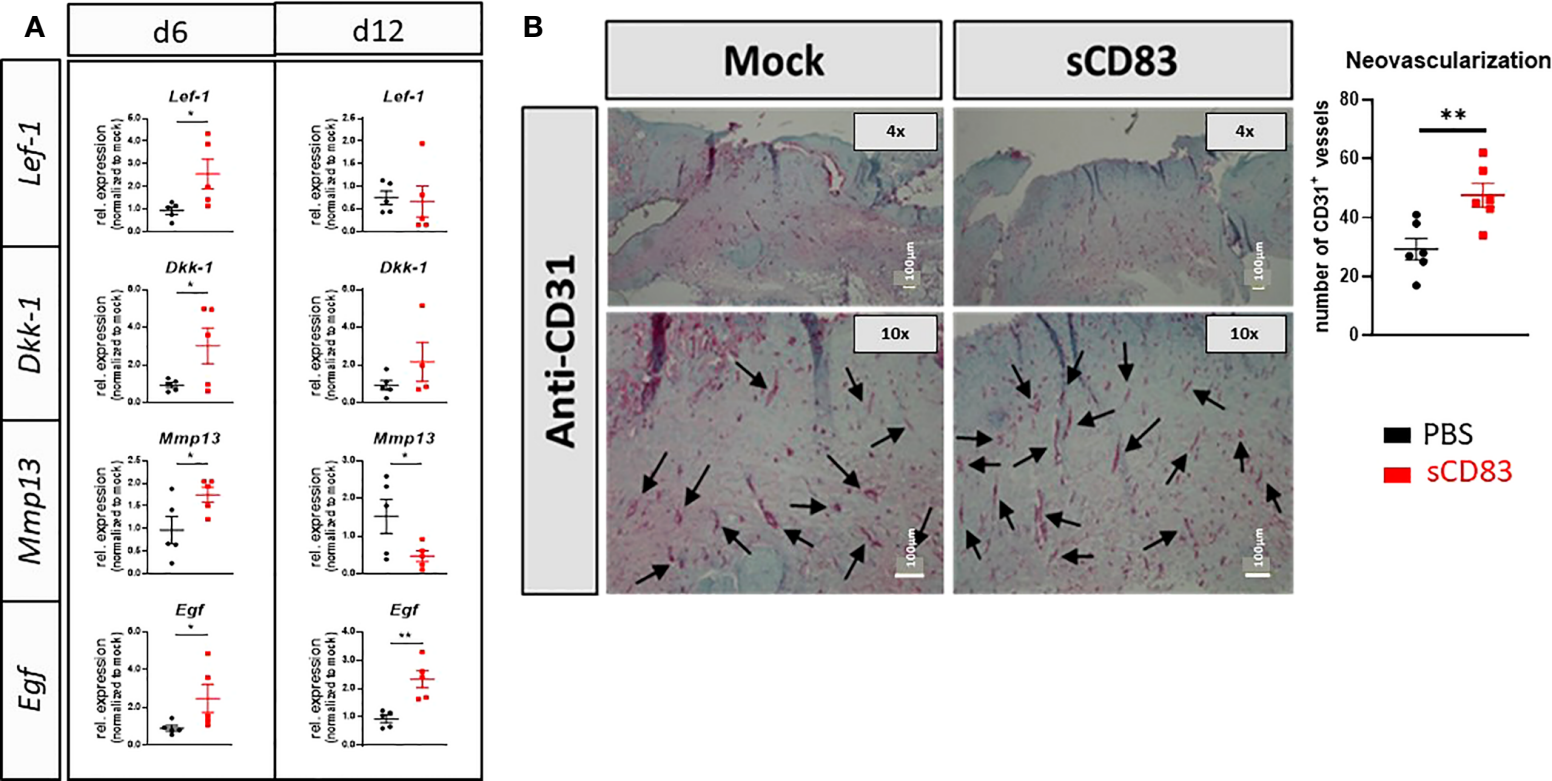
sCD83 promotes epithelial stem cell activity during wound healing processes. **(A)** RT-PCR analyses were performed on day 6 and day 12 using wound biopsies from sCD83- or mock-treated mice with n≥5. **(B)** Blood vessel formation was assessed by immunohistochemical stainings, using a CD31-specific antibody. Vessel are highlighted by arrows along with the graphical representation of CD31^+^ vessels right-hand side; n=6). Data are illustrated as mean ± SEM. Statistical analyses were performed using Mann-Whitney test. Asterisks mark statistically significant difference (*p< 0.05 and **p< 0.01). The absence of asterisks indicates that there is no statistical significance.

In order to investigate and proof a possible involvement of the Wnt/Frizzled pathway, which was found to be upregulated in our RNA sequencing analyses by sCD83 (**Figure 2**), we analyzed the expression of two signature genes of WNT signaling, *Lef-1* and *Dkk-1* by RT-PCR on day 6 and 12. In sCD83-treated mice a significant induction of Lef-1 and Dkk-1 was detectable on day 6, but not on day 12. Thus from these data we conclude, that stem cell activity might no longer be required for wound healing in sCD83-treated mice, at later time points. (**Figure 4A**) (39). Concomitantly, we observed increased expression levels of the specific growth factor *Egf* on day 6 and 12, indicating ongoing epithelial and vascular remodeling in the closed wounds (40–42). In addition, expression of *Mmp13*, which has been reported to stimulate migration of fibroblasts and keratinocytes during the formation of granulation tissue (39, 43), was significantly upregulated in sCD83-treated animals on day 6 (**Figure 4A**). However, on day 12, *Mmp13* mRNA levels were significantly downregulated when compared to mock controls (**Figure 4A**). In summary, these transcript-profiles point towards accelerated stem cell proliferation and the formation of granulation tissue around day 6, which is accomplished on day 12 in sCD83-treated mice, but not the control animals. However, the continuous expression of *Egf* drives stem cells towards differentiation, e.g. into blood vessels, which further support wound healing processes and tissue repair, (**Figure 4A**). This is supported by our immunohistochemical analyses showing significantly increased numbers of CD31-positive blood vessels in skin biopsies derived from wounds of sCD83-treated mice (**Figure 4B**). Of note, ingrowth of blood vessels into the wound sites is rather a part of the wound remodeling phase and thus remarkably accelerated in sCD83-treated samples (44). Of note, hair growth and/or hair follicle formation, as observed in our RNA sequencing analyses (**Figure 2D**) is intimately associated with blood vessel formation (45) and normally absent from wound healing of small wounds (46). Induction of hair growth requires epithelial and mesenchymal stem cell activation driven by Wnt and Egf concomitantly with a timely decrease of respective inhibitors such as Dkk-1 (47–49).

### Topical administration of sCD83 on wounds efficiently accelerates wound closure

**Figure 5:**
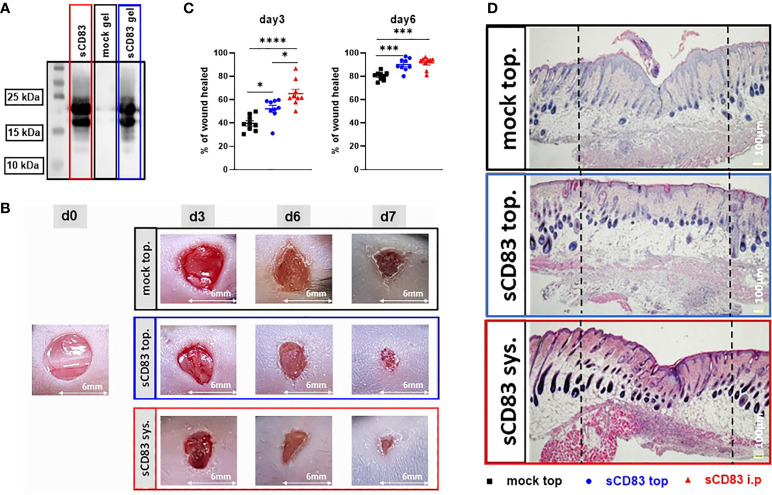
Topical sCD83 application induces local wound healing. Wound lesions were induced using 6 mm punches, and diameters were measured by caliper, at indicated time points. **(A)** Western blot analyses of sCD83-hydrogel matrices after 10 days at 4°C, demonstrate the stability of the sCD83 protein. **(B)** Daily topical administration of sCD83 on days 1, 3 and 5 significantly increased wound healing. **(C)** Percentage of wound closure was set relatively to the day 0 value, with each group n=10. **(D)** Representative histological analyses of day 7 samples. Dashed lines mark the former wound sites. Data are illustrated as mean ± SEM and statistically analysed using One-way ANOVA for C. Asterisks mark statistically significant difference (*p < 0.05, ***p < 0.001 and ****p < 0.0001). The absence of asterisks indicates that there is no statistical significance.

Regarding a possible future application of sCD83 for the acceleration of human wound healing, a topical application would be of high clinical value. Obvious advantages of topical formulations include easier handling as well as reduced systemic side effects, when compared to systemic internal administration. Thus, we next investigated whether a topical application, directly onto the wound bed, would also induce an accelerated wound healing, as observed using the systemic route and in the cell transfer experiment. For this purpose sCD83 was mixed into a hydroxyethyl-cellulose gel and used for subsequent topical application experiments. First, we analyzed the stability of the sCD83 molecule in this particular gel over a time period of 10 days, at 4°C, and observed that sCD83 was stable within the assessed time period (**Figure 5A**). Subsequent *in vivo* experiments showed, that the daily topical administration of 25 µg sCD83 per wound site on days 1, 3 and 5 significantly increased wound healing on day 3 and day 6 (**Figure 5B**). Both, the systemic as well as the topical sCD83 application revealed an advanced wound healing process on day 3, in comparison to control treated mice. However, at this time point the systemic route yielded slightly better results (**Figure 5C**, left). This was no longer the case on day 6, whereby both topically- and systemically sCD83 treated mice showed the same highly significantly improved wound healing features, when compared to control mice (**Figure 5C**, right). Histological analyses of day 7 cutaneous wound biopsies confirmed the advanced stage of wound healing in topically sCD83-treated mice, as indicated by thinning of the epidermis layer and the presence of hair follicles at the former wound site (**Figure 5D**). In contrast, in mock treated controls we did not observe a complete regeneration of epidermal and dermal tissue, indicating an incomplete wound healing status. From these data we conclude that both, the systemic as well as the topical sCD83 administration route induce a fast and complete wound healing process. For a future clinical application the topical route has distinct advantages.

## Discussion

In several studies the immunomodulatory potential of sCD83 in the context of autoimmune disorders and transplantation has been demonstrated (6). Amongst others, administration of sCD83 ameliorated disease progression in murine autoimmune models such as experimental autoimmune encephalomyelitis (EAE), systemic lupus erythematosus (SLE), inflammatory bowel disease (IBD) and antigen-induced arthritis (AIA) (6, 15, 24, 25, 50). Analogously, administration of sCD83 significantly improved graft survival in transplantation models of cornea, heart, kidney, and skin, which depended on the IDO1-mediated induction of Treg differentiation and proliferation (9, 11, 51–53). In the present study, we report for the first time the pro-regenerative effects of sCD83 on cutaneous wound healing. These results fundamentally expand our knowledge regarding the functions of sCD83 and further highlight the immunomodulatory potential of this molecule. Moreover, the capacity to promote wound healing as well as hair growth after topical application holds great promise for the promotion of functional wound healing in clinical care.

In order to investigate whether sCD83 treatment interferes with early cellular wound healing processes, we have analyzed the cellular composition within the wound beds of sCD83-treated mice and observed a significant reduction of neutrophils in the skin. In contrast, the numbers of monocytes and macrophages increased, initiating the transition towards the proliferation phase of the wound healing process (54). In addition, a significant accumulation of B and T lymphocytes was present in sCD83-treated mice. Statistical analyses were based on the approved number of individual animals. It has been reported that wound closure is accelerated in the presence of mature B cells, and T cell function is indispensable for wound closure (55, 56). In this respect we show that sCD83 modulates the initial inflammatory wound healing phase as well as the subsequent anti-inflammatory proliferation- and remodeling phases (**Figures 2**-**4**). Neutrophils and macrophages represent the dominant pro-inflammatory cell population during the early stage of wound healing and regulate the local and systemic defense of the wound (57). Neutrophils secret amongst others high amounts of proteases, reactive oxygen species, and pro-inflammatory cytokines to sterilize the wound area (58). At this stage, macrophages differentiate into an inflammatory phenotype, characterized by the phagocytic activity to clear wound debris and further stabilize the inflammatory microenvironment (59). Once the inflammation phase is completed, neutrophils undergo apoptosis and are phagocytosed by macrophages in a process termed efferocytosis (60). In this context, efferocytosis activates a “feed-forward pro-resolution program” in macrophages, which undergo a subsequent trans-differentiation into a IL-10 producing, pro-resolving phenotype (61).

With respect to the early stage of wound healing, RNA sequencing analyses revealed that overall ~2.000 gene transcripts were significantly up- or down-regulated as early as day 3 after wound healing and that numerous wound healing-associated pathways and transcripts were associated with the sCD83 treatment. In this context, a very interesting finding was, that the process of wound healing was strongly upregulated in sCD83-treated mice, as indicated by specific *Wnt* signaling transcripts, which positively affect wound healing processes (62). Of note, the pathway termed “response to wounding” was represented among both, the top upregulated- as well as top downregulated pathways (**Figure 2B**), which at the first view sounds contradictive. However, a closer look at transcriptome-level revealed that upregulated transcripts in the “response to wounding” that were upregulated included *Wnt* and *Notch*. TNFα is also upregulated within this pathway, and according to *Ritsu* and colleagues, this is critical during the early processes of wound healing (63). In contrast to inflammatory autoimmune disorders, where the pathogenesis is based on an excessive and long lasting pro-inflammatory cytokine secretion, a short but intense inflammatory immune response, strongly accelerates wound healing processes (64). The important role of TNFα during early processes of wound healing was further confirmed by their blockade, either by knockout or depletion, resulting in an inefficient wound healing (63). In contrast, downregulated “response to wounding” included transcripts of early (inflammatory) wound healing such as *Ecrg4* (**Figure 2C**), which is required for proper neutrophil recruitment (27). Moreover, proteins such as Acta2 in myofibroblasts contribute to the wound healing process, but are dispensable for proper wound closure (65). Of note, Acta2^+^ myofibroblasts rather contribute to the onset of fibrotic wound healing when not suppressed in time (66). In sharp contrast, upregulated transcripts in the “response to wounding” included transcripts such as *Wnt* and *Notch*, known to be important during wound healing (67). In addition, *Cd109, Cd44* and *Klf4* were also significantly upregulated (**Figure 2C**). While CD109 contributes to increased wound healing quality by antagonizing fibrotic wound healing, KLF4 promotes the trans-differentiation process from inflammatory MΦ into their pro-resolving tissue repair phenotype (68, 69). Likewise, CD44 contributes to the resolution of the inflammatory phase and the progression towards the proliferation phase, since its depletion results in excessive inflammation and a low quality of the newly formed skin (70).

Of particular note, using the IPA algorithm, pathways associated with hair follicle formation were also predicted to be significantly upregulated by sCD83 (**Figures 2B, D**). Hair follicle formation is known to depend on epidermal and fibroblast stem cells activation, which is not only beneficial for accelerated wound healing (71, 72), it also indicates a more functional wound healing process as it associates with blood vessel neogenesis and decreased scar formation (32, 45). This further corresponds to another surprising finding in the transcriptome analyses of sCD83-treated wounds, which revealed an association of wound healing mechanisms with embryonic morphogenesis in which wound healing occurs with complete formation of unscarred skin, epidermis and hair follicles (**Figure 2B**). Of note, fetal wound healing is regarded as an optimal process of tissue regeneration, which to date could not be achieved in adults (73).

With respect to the sCD83-induced pro-regenerative effects, we identified MΦs as pivotal players in this setting. MΦs coordinate the wound healing process throughout the inflammatory, proliferative and remodeling phase, e.g. by the clearance of apoptotic debris, orchestrating the resolution of inflammation, promoting fibroblast and keratinocyte activity as well as the remodeling processes (74). RT-PCR analyses suggested the induction of pro-resolving tissue repair MΦs in sCD83-treated wounds, as increased levels of signature transcripts such as *Tnfα, Ym-1* and *Il-10* were observed (75). In addition, MΦs represent a major source of SLPI, a molecule involved in resolution of inflammation, since SLPI knockout mice suffered from impaired wound healing and delayed resolution of inflammation (34, 76). Interestingly, although sCD83 treatment was continued until day 7, the expression of pro-inflammatory cytokines started to decline already before day 6 compared to mock controls. Again, indicating a speeding up throughout the healing process, which does not simply rely on boosting IL-1β and TNFα expression but also on an earlier induction of factors disinhibiting epithelial proliferation in epidermis and hair follicle such as *Dkk-1* (49). This multifactorial modulation by sCD83 is further supported by the stabilized expression of YM-1, a key marker for tissue repair macrophages, which is vital for the subsequent transition towards proliferation and reepithelization (77).

To further analyze the effects of sCD83 on MΦ, which also express the sCD83 ligand, i.e. the TLR4/MD-2 receptor complex, we investigated the differentiation process towards a pro resolving MΦ phenotype and compared sCD83 alone or in combination with IL-4. A very prominent induction of *Tnfα, Il-10, Vegf and Msr-1* were observed in combination with IL-4, indicating the induction of a pro-resolving MΦ-associated phenotype. To analyze, if this sCD83 induced pro-resolving phenotype is of relevance also *in vivo*, adoptive transfer experiments were directly performed onto the wounds of mice, comparing sCD83-pretreated MΦ, untreated MΦ or PBS. In this *in vivo* setting, no additional IL-4 was applied to wound sites, as IL-4 naturally accumulates shortly after wound infliction and thus allows the analyses of sCD83-mediated effects during regular cutaneous wound healing (38). Strikingly, transfer of sCD83-pretreated MΦs resulted in a highly and significantly accelerated wound closure on day 3. In contrast, untreated MΦ did not improve wound healing, which is in line with the work reported by Jetten *et al.* (78). Therefore, we conclude that sCD83 does not only induce the polarization and trans-differentiation of MΦs, but in addition boosts the remodeling activity of MΦ throughout the wound healing process.

The RNA sequencing analyses of wound samples on day 3 further revealed a prominent role of the Wnt/Frizzled axis in the sCD83-treated mice. Thus, we tracked the induction of typical signature transcripts, e.g. *Lef-1* and *Dkk-1*, and found both significantly upregulated on day 6 (39). Noteworthy, *Lef-1* expression is closely associated with an improved skin regeneration as well as hair regrowth, which is an indicator for a high quality wound healing process (79, 80). Of interest, also transcripts downstream the Wnt cascade, i.e. *Mmp13* and the growth factor *Egf* were upregulated 6 days post wounding, indicating a co-activation of alternative pathways by sCD83, such as the GHOST response, reported by Doumpas and colleagues (39). In the context of wound healing, *Mmp13* and *Egf* provide crucial pro-regenerative signals, since their administration resulted in a strikingly accelerated wound closure (81, 82). Of note, the administration of growth factors could be an interesting approach in the field of wound care, but due to their short half-life *in vivo*, it is not applicable in practice (83). Thus, sCD83-induced regenerative mechanisms bypass these limitations via the direct induction of growth factors at the *locus operandi*, as exemplary shown by the increased numbers of blood vessels in sCD83-treated mice.

In view of a possible clinical application, the finding that sCD83 significantly improved cutaneous wound closure also as topical application, is very encouraging. Although to date no severe side effects were reported upon systemic sCD83 application in preclinical *in vivo* models, a locally-restricted medication has definitively great advantages. Of note, a recent study, where we have incubated corneal grafts with labeled-sCD83 prior to transplantation, showed cell-associated sCD83 within the recipient’s eye draining lymph nodes, thereby highlighting the modulatory capacities of sCD83 beyond local restrictions (14).

**Figure 6:**
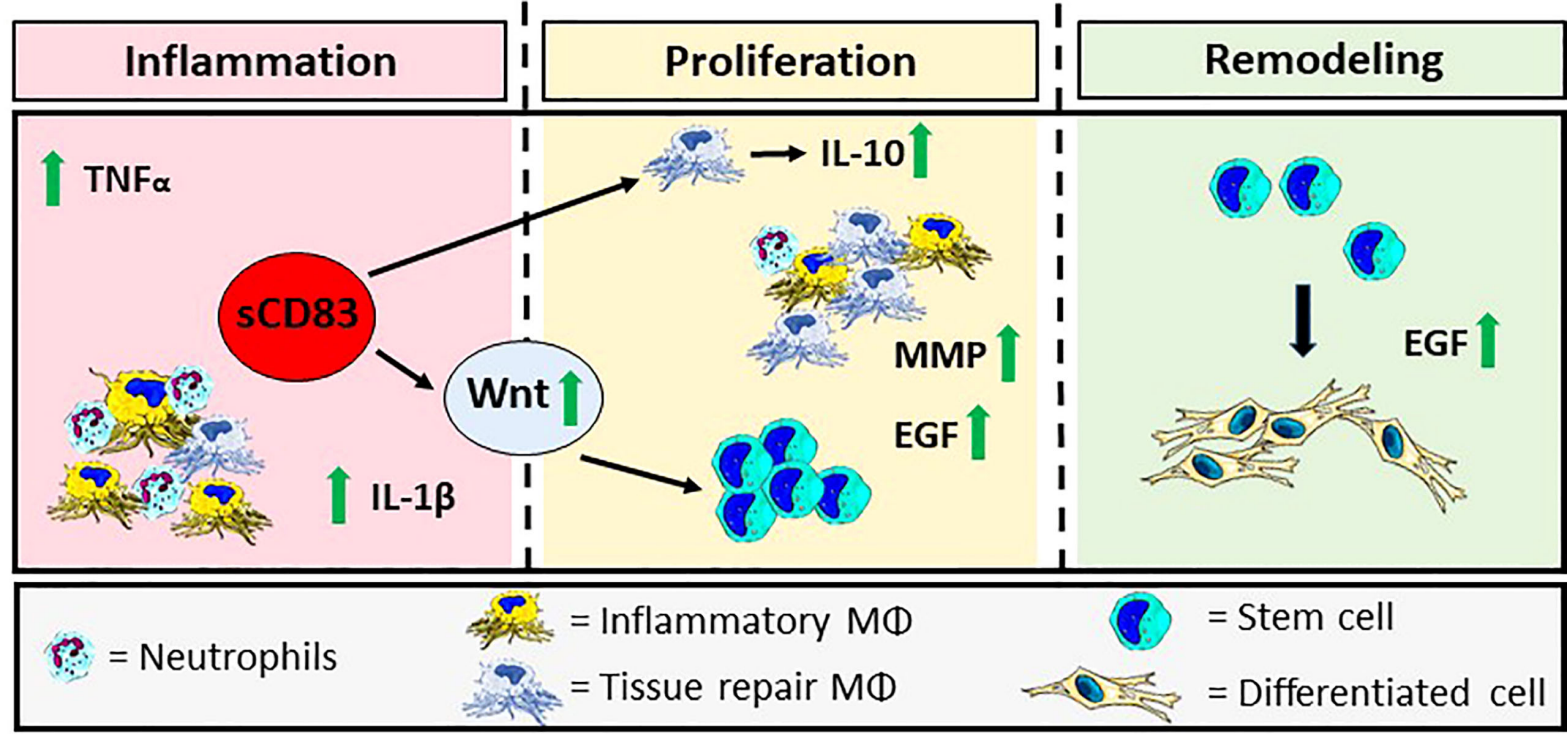
Graphical Overview: sCD83-treatment accelerates cutaneous wound healing modulating the three phases of wound closure. sCD83 boosts the natural kinetics of the inflammatory-, proliferative- and remodeling phases, during the wound healing process. Initially, the inflammatory phase, within wound sites of sCD83-treated mice, was increased and prolonged, as indicated by upregulated TNFα and IL-1β levels, while IL-10-expression was delayed, until the proliferative phase. During wound healing YM-1^+^ sCD83 induced resolving MΦ are responsible for the secretion of IL-10 and the resolution of inflammation. Concomitantly, growth factors such as EGF and MMP remodeling enzymes are released by alternatively activated MΦ in order to restore tissue function and quality. Furthermore, the canonical Wnt-signaling cascade, which is related to epithelial stem cell proliferation, is boosted at earlier time points in order to replace the ablated tissue. The biological consequences are reflected in the increased presence of blood vessels in wounds of sCD83-treated mice.

Finally, a graphical summary is depicted in **Figure 6**, which visualizes the sCD83-induced effects during the inflammatory-, proliferative-, and remodeling phases of wound healing. The presence of sCD83, during the initial phase boosts the expression of pro-inflammatory mediators, such as TNFα and IL-1β. Subsequently, IL-10 expressed by sCD83-induced resolving MΦ not only resolves the inflammatory phase, but provides further stimulating effects regarding angiogenesis and extracellular matrix remodeling, resulting in a significantly accelerated wound healing (84, 85).

In summary, our data show that via the induction and reprogramming of MΦ, sCD83 modulates different stages of wound healing, thereby accelerating and improving wound healing.

## Data availability statement

The original contributions presented in the study are publicly available. This data can be found here: GEO bank: GSE214079.

## Ethics statement

The animal study was reviewed and approved by Goverment of Unterfranken, Würzburg

## Author contributions

DR, EZ, KP-M and AS designed the experiments, evaluated the results and wrote the manuscript. DR, EZ, CK, KP-M, LS, AW, MT, MF, MK, and CO acquired data. DR, EZ, AS, CK, KP-M, LS, AW, MT, CO, FK, MF, MK, ES, EP, SE, and CB interpreted data and edited the manuscript. SS and GL prepared sCD83-hydrogels for topical application. AS, DR, EZ, CO, and SE acquired funding. All authors contributed to the article and approved the submitted version.

## Funding

This work was supported by the Deutsche Forschungsgemeinschaft (DFG) within grants SFB1181 project B03 and STE432/15-1 (to AS), ZI 1225/1-1 (to EZ), OS 578/2-1 (to CO), FOR2240 project P5, EM48/5-2 (to SE) and the Interdisciplinary Center of Clinical Studies (IZKF) at the University Hospital of the FAU Erlangen-Nuremberg (ELAN P077) (to DR). KP-M was funded by the Bavarian Equal Opportunities Sponsorship—Realization Equal Opportunities for Women in Research and Teaching, and German Federal Ministry of Education and Research (BMBF) CompLS program grant 031L0262C (to MK) and Fraunhofer Cluster of Excellence Immune-Mediated Diseases (CIMD; to MF and MK).

## Conflict of interest

The authors declare that the research was conducted in the absence of any commercial or financial relationships that could be construed as a potential conflict of interest.

## Publisher’s note

All claims expressed in this article are solely those of the authors and do not necessarily represent those of their affiliated organizations, or those of the publisher, the editors and the reviewers. Any product that may be evaluated in this article, or claim that may be made by its manufacturer, is not guaranteed or endorsed by the publisher.
