# Supplementary material for: Soluble CD83 improves and accelerates wound healing by the induction of pro-resolving macrophages
Source: Front Immunol. 2022 Sep 30;13:1012647. doi: 10.3389/fimmu.2022.1012647 (PMC9564224; doi:10.3389/fimmu.2022.1012647)
Supplement: Supplementary file 1 [file DataSheet_1.docx]

Supplementary Material

## Supplementary Figures


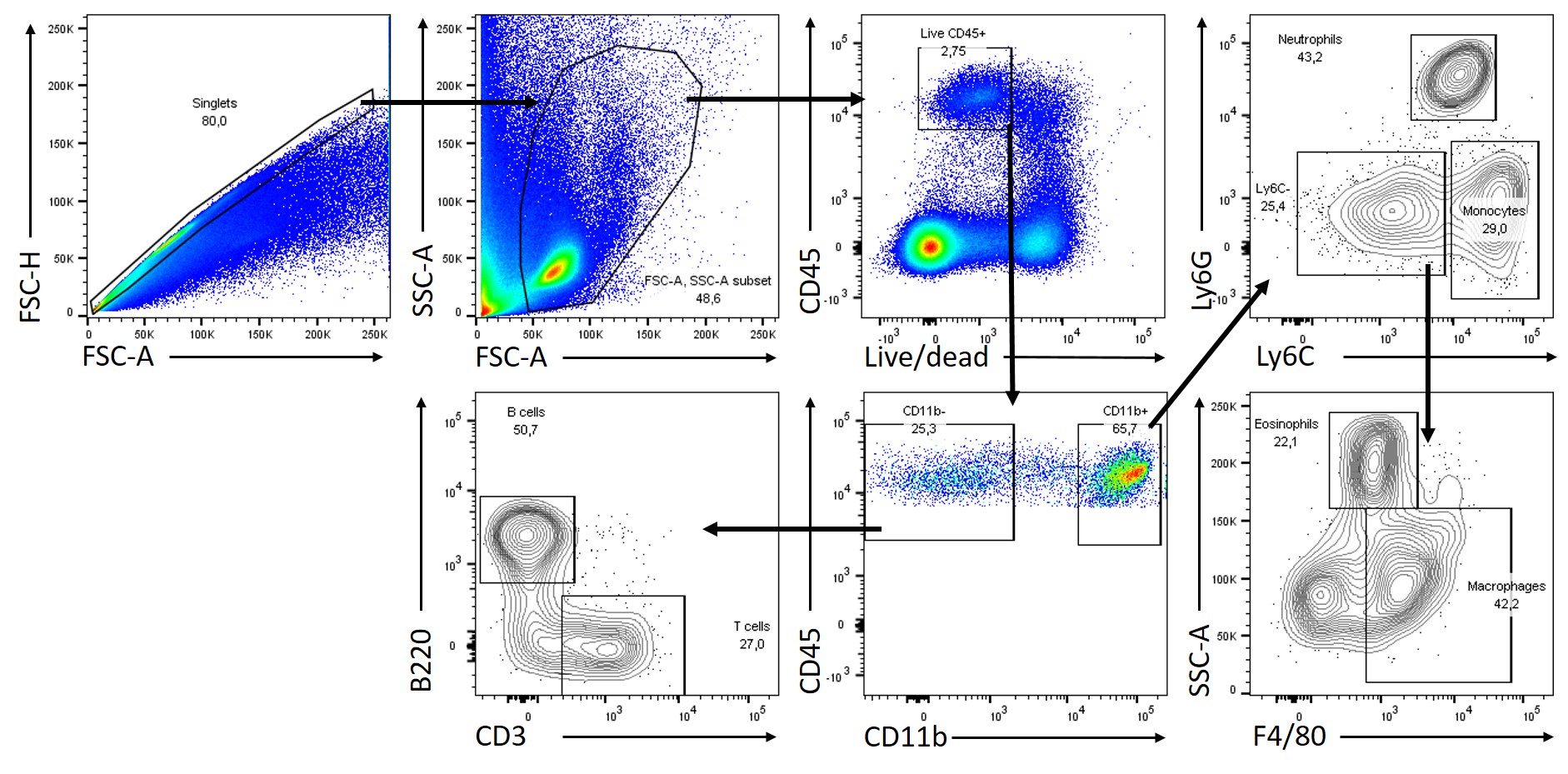


Supplementary Figure 1. Gating strategy for the flow cytometric analyses of skin biopsies.

After cell extraction from murine skin biopsies, the cells were stained using fluorescence-coupled antibodies and LIVE/DEAD Fixable Aqua DEAD Cell stain to exclude dead cells from analysis. Experiment was performed using the FACS Canto II flow cytometer.


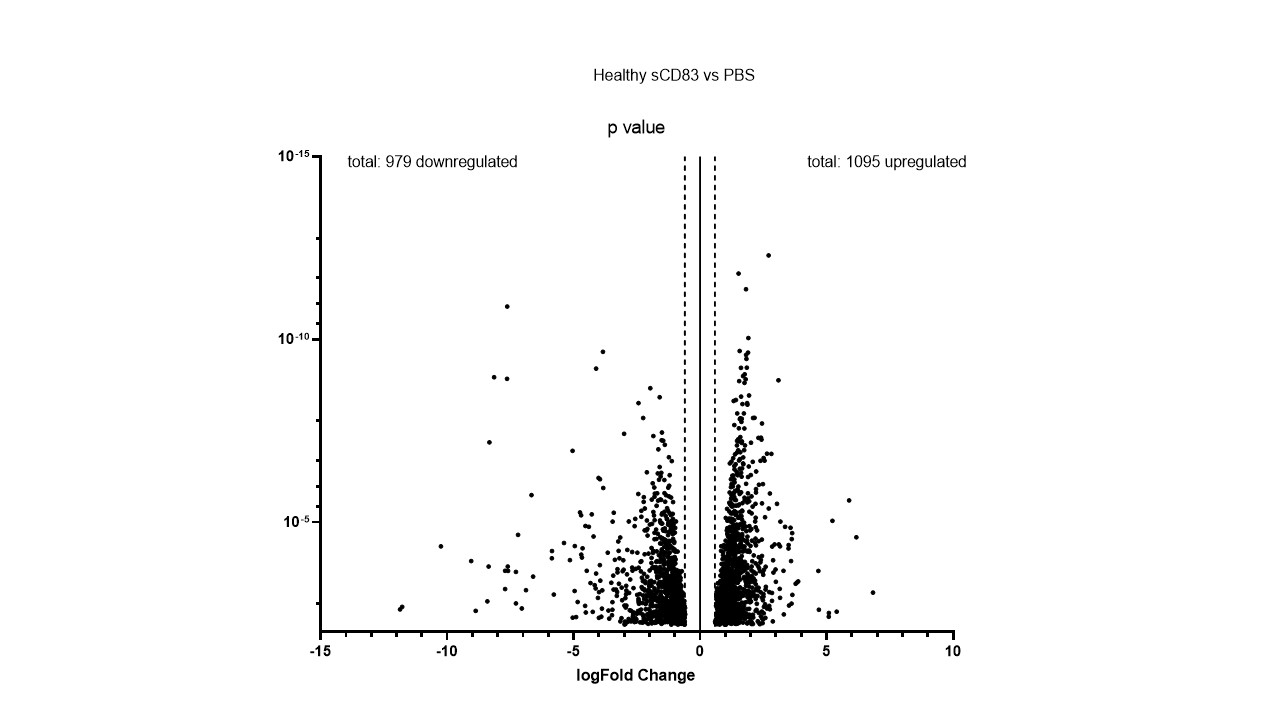


**Supplementary Figure 2.** sCD83 strikingly modulates the wound healing process three

days after wound infliction. Wound biopsies were isolated three days after wound infliction

from mock- or sCD83-treated mice and RNA was isolated for RNA sequencing analyses.

LogFC (x-axis) and p value (y-axis) were determined using R software.
